# A Devastating Case of Hepatitis C-Induced Mixed Cryoglobulinemia

**DOI:** 10.1155/2021/8244432

**Published:** 2021-10-08

**Authors:** Sameeha Khalid, Dhuha Alhankawi, Kamalmeet Kaur, Ali Ali, Anna Kazaryan, Marina Roytman

**Affiliations:** ^1^Department of Internal Medicine, University of California San Francisco-Fresno, Fresno, CA, USA; ^2^Department of Gastroenterology & Hepatology, University of California San Francisco-Fresno, Fresno, CA, USA; ^3^Department of Rheumatology, University of California San Francisco-Fresno, Fresno, CA, USA

## Abstract

Hepatitis C-induced mixed cryoglobulinemia leading to rapidly progressive gangrene, necessitating amputations, is a rare presentation. We describe a case of a 55-year-old man with untreated chronic hepatitis C virus (HCV) presenting with arthralgia and palpable purpura, which rapidly progressed to life-threatening gangrene of all extremities requiring amputations in the setting of mixed cryoglobulinemia. Treatment for HCV was initiated which led to the arrest of gangrene progression and the patient's survival. Patients with HCV-induced cryoglobulinemia should be closely monitored and started on early therapy with direct-acting antiviral therapy to prevent progression of vasculitis to gangrene. Universal screening for HCV can aid in early diagnosis and treatment to prevent devastating consequences.

## 1. Introduction

Mixed cryoglobulinemia is a rare condition, with a prevalence of 1 : 100,000 in the United States. The most common cause is chronic hepatitis C virus (HCV); however, a number of other etiologies including other infections, autoimmune diseases, and lymphoproliferative disorders are known to precipitate mixed cryoglobulinemia [1]. Meltzer coined the clinical triad of mixed cryoglobulinemia as palpable purpura, arthralgia, and weakness in 1966; however, it is unlikely for patients to present with all three, and palpable purpura is the most common presentation [2].

Chronic HCV infection is known to be the underlying disorder in a majority (80–90%) of cases of mixed cryoglobulinemia; however, only 5–10% of HCV patients are estimated to have symptomatic disease [2, 3]. In many cases, the extrahepatic manifestations of HCV can occur in patients without features of overt liver disease. The role played by HCV in the development of this disease is thought to stem from chronic stimulation of the immune system causing B-cell hyperactivation and selective expansion of cryoglobulin-producing B-cell clones which express rheumatoid factor (RF) and proliferate in the liver, serum, and lymph nodes [4, 5]. Circulating cryoglobulins precipitate at low temperatures below 37 degrees Celsius and deposit in small and medium vessels, leading to complement activation and inflammation and subsequently causing systemic vasculitis and damage to various end organs [4]. It is the circulation of these large cryoproteins that leads to increased blood viscosity causing hypoperfusion and subsequently gangrenes [2].

There is a wide spectrum of manifestations associated with cryoglobulinemia which can range from mild (>50%) to moderate or severe (35%) and even life-threatening conditions (<15%) [6]. Life-threatening cryoglobulinemia vasculitis can be diagnosed if any of the following are present: renal involvement, gastrointestinal involvement, lung involvement, CNS involvement, heart involvement, widespread vasculitis (multiorgan involvement), hyperviscosity syndrome, or liver failure in HCV-related mixed cryoglobulinemia [3]. Such life-threatening features carry a mortality rate between 20% and 80% and in almost two-thirds of cases may be the initial clinical manifestation of cryoglobulinemia [6]. HCV-induced mixed cryoglobulinemia leading to rapidly progressive gangrene of all four extremities, necessitating amputations, is a rare presentation of cryoglobulinemia that we present here.

## 2. Case Report

**Figure 1:**
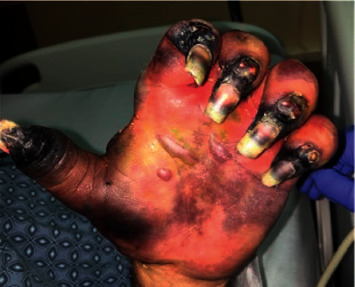
Patient's hand on initial presentation.

**Figure 2:**
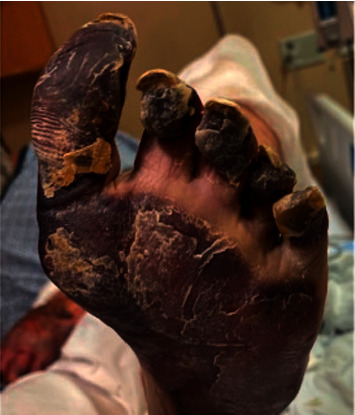
Patient's foot on initial presentation.

A 55-year-old man with treatment-naive HCV infection (genotype 1a) presented to the emergency room with bilateral hand pain and diffuse purpuric rash. He described associated arthralgias, purpuric lesions, weakness, and numbness. Physical examination showed palpable purpura and extensive gangrene affecting bilateral upper and lower extremities (Figures 1 and 2). Laboratory investigation revealed positive rheumatoid factor (>1,200 IU/mL), hypocomplementemia (C4 <8.0 mg/dL), HCV RNA 2,390,000 IU/mL, and positive cryoglobulins. Skin biopsy of the leg showed mixed, predominantly acute, infiltrate with scattered small to mid-sized vessels with features of fibrin thrombi with or without associated vasculitis. The patient was diagnosed with mixed cryoglobulinemia complicated by severe cutaneous vasculitis with ischemic limbs.

**Figure 3:**
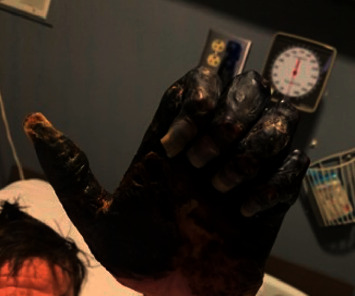
Progression of gangrene 10 days after initial presentation.

**Figure 4:**
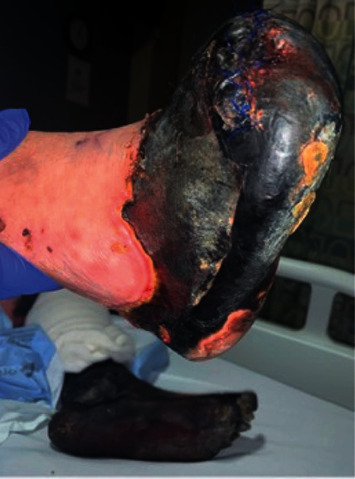
Bilateral gangrenous lower extremities 10 days after initial presentation.

Plasmapheresis was initiated; however, gangrene continued to progressively worsen despite it (Figures 3 and 4). He was transitioned to immunosuppressive therapy with high-dose pulse steroids followed by prednisone 60 mg daily for four weeks in combination with rituximab without improvement. Given progressive gangrene despite multiple surgical debridements, inpatient treatment with sofosbuvir/velpatasvir was started for treatment of chronic HCV infection. After initiation of HCV treatment, the viral load went from 2,390,000 IU to <15 IU after four weeks and there was no further spread of gangrene. The necrotic limbs were unsalvageable, requiring serial limb amputations including bilateral above the knee amputations, right metacarpophalangeal disarticulation of fingers, right interphalangeal disarticulation of the thumb, and left wrist disarticulation. He was discharged to a skilled nursing facility. On follow-up with Hepatology, the patient achieved sustained virologic response week 12 (SVR-12) and has not had further recurrence of manifestations associated with mixed cryoglobulinemia.

## 3. Discussion

There is a wide spectrum of manifestations associated with cryoglobulinemia with severe and life-threatening presentations being relatively rare (<15% of cases) [6]. Most patients with such severe presentations do not survive. One case study by Manuel Ramos-Casals, MD, of 29 patient cases demonstrated a 66% mortality in patients presenting with severe, life-threatening cryoglobulinemia vasculitis [7]. It remains unclear why some patients manifest a severe form of cryoglobulinemia vasculitis, and information regarding clinical presentation and prognosis of these patients is limited. Our patient with HCV-related mixed cryoglobulinemia presented with severe cutaneous vasculitis progressing to gangrene of all extremities with the catastrophic result of unsalvageable limbs. Upon literature review, this is one of the most aggressive cases of mixed cryoglobulinemia vasculitis reported thus far that resulted in the patient's survival. There have been no other cases reported that have resulted in multiple limb amputations secondary to this extent of cryoglobulinemia. As such, this case serves to increase clinician awareness of the severe gangrene that may rapidly develop in mixed cryoglobulinemia. These cases require early recognition and aggressive treatment to prevent progression to the extent of limb amputation and even death.

The mainstays of treatment of HCV-induced mixed cryoglobulinemia include the eradication of HCV infection, deletion of underlying B-cell clonal expansions, and depletion of cryoproteins [1, 8]. Multiple studies agree that regardless of the severity of mixed cryoglobulinemia, an attempt to eradicate HCV should be pursued whenever possible and as soon as possible as suppression of viral replication may limit or halt the immunopathogenic process triggered by HCV [9, 10]. In the presence of life-threatening manifestations of mixed cryoglobulinemia, it is vital to begin rapid treatment with immunosuppressive therapy concomitantly with direct-acting antiviral (DAA) therapy. A prospective study showed that DAA therapy is highly effective and safe for patients with HCV-induced mixed cryoglobulinemia with an overall 100% clinical response rate for vasculitis [11].

This case notably highlights the necessity of universal screening for HCV and portrays the devastating effects that delayed screening and therefore delayed treatment may have. Universal screening is imperative for the early detection, treatment, and prevention of the intrahepatic and extrahepatic consequences of HCV, such as mixed cryoglobulinemia.

## Data Availability

No data were used to support this study.
